# Selected aspects of the psychosocial functioning of medical personnel of emergency medical teams—a cross-sectional study

**DOI:** 10.3389/fpubh.2025.1754424

**Published:** 2026-01-13

**Authors:** Jakub Bartoszuk, Matylda Sierakowska

**Affiliations:** Faculty of Health Sciences, Medical University of Bialystok, Bialystok, Poland

**Keywords:** coping strategies, paramedic, psychosocial functioning, self-efficacy, stress

## Abstract

**Introduction:**

A paramedic is one of the most mentally demanding professions, requiring responsibility for the health and lives of patients, quick decision-making, and contact with suffering and death. Due to the unpredictable nature of their line of work, this professional group faces high levels of stress. Identifying protective factors, such as personal resources, self-efficacy, and commitment to work, helps better understand the mechanisms of coping in difficult situations and enables corrective and preventive measures to be taken. The aim of the study is to identify and analyse in detail occupational stress among emergency medical service workers, in relation to respondents’ psychological resources, work engagement, sense of self-efficacy, and strategies for coping with difficult situations.

**Research methodology:**

The survey, delivered online via Google between May and September 2025, targeted paramedics and nurses working at the Provincial Ambulance Service in Białystok (north-eastern Poland). A diagnostic survey method was used, employing a proprietary questionnaire and standardised tools such as the Resilience Measurement Scale (SPP-25), the Utrecht Work Engagement Scale (UWES), the General Self-Efficacy Scale (GSES), the Perceived Stress Scale (PSS-10), and the Inventory for Measuring Coping with Stress (Mini-COPE).

**Results:**

The mean age of the respondents (*n* = 150) was 43.17 years. The study group consisted of 51.3% women and 48.0% men. The majority of respondents had a master’s degree in nursing (42.7%) or a bachelor’s degree in emergency medicine (32.0%). The study revealed that respondents reported high levels of stress (PSS-10 score of 22.31), moderate self-efficacy (GSES score of 27.6), moderate work engagement (UWES score of 3.52), and low psychological resilience (SPP-25 score of 64.95). In terms of coping strategies (Mini-COPE), respondents most often sought emotional and instrumental support and relied on humour and planning strategies. The results indicated significant negative correlations between age and length of service (*r* = −0.58; *p* < 0.001; rho = −0.52; *p* < 0.001) with psychological resilience, with overall commitment to work (*r* = −0.56; *p* < 0.001; rho = −0.61; *p* < 0.001) and negative correlations between age and length of service with self-efficacy (*r* = −0.43; *p* < 0.001; rho = −0.41; *p* < 0.001). The number of hours worked was negatively correlated with perceived stress (*r* = −0.26; *p* < 0.01). Women scored significantly higher on overall psychological resilience (*p* = 0.003) and work engagement (*p* = 0.002), while perceived stress levels were significantly higher among women than men (*p* = 0.006).

**Conclusion:**

The respondents were characterised by moderate to high perceived work stress, low psychological resilience, and moderate work engagement and self-efficacy. Dominant coping strategies included emotion-focused, problem-focused, and adaptive strategies. Older individuals with longer work experience had lower levels of psychological resources. Respondents differed by gender in terms of their psychological resources and work engagement.

## Introduction

Stress is unavoidable and is part of everyone’s life, regardless of age, education, or profession. It causes an emotional and psychological imbalance. A number of factors influence perceived stress, which, in turn, directly affects quality of life and life satisfaction. And although everyone encounters stressful situations in their daily lives and seeks ways to cope, some are particularly exposed to stress due to their profession. Emergency medical team (EMT) personnel are among the professional groups for whom stress is an inherent part of their work.

By definition, stress is a negative feeling, a physiological reaction of the body to a threatening situation. Evolutionarily, stress, as a state of heightened tension, is intended to mobilise the body in situations that threaten one’s life or limb. Its purpose is to enable the survival of humans as a species. In the 1950s, Hans Selye was the first to discuss stress from a scientific perspective, drawing attention to its psychological effects and related emotions, such as anger, fear, and sadness, as well as behavioural responses, such as aggression, crying, and withdrawal (1, 2). Walter Cannon developed a concept according to which the response to a stressor involves maximum concentration and increased tension, intended to mobilise all the body’s energies to either confront the problem or flee (1, 3). Researchers studying stress emphasise that individuals who are exhausted and have fewer resources (sleep, well-being, etc.) cope less well in stressful situations. In addition, greater stress is experienced by individuals exposed to external (environmental) factors (e.g., poor atmosphere at work, bullying, heavy workload, low pay, monotony, shift work, hazardous working conditions, etc.) or internal (person-specific) factors (e.g., mental disorders, depression, addictions, female gender, lack of support, low self-esteem, chronic pain, etc.), which may result from psychosocial conditions, private life, or the environment (2, 4–10).

Stress, especially chronic stress, is very harmful. It not only affects well-being and quality of life, but also often predisposes to the development of somatic diseases, including autoimmune ones. It may deteriorate mental health, causing mental disorders or exacerbating existing psychiatric conditions. In some cases, it even affects the deoxyribonucleic acid (*DNA*). It generally disrupts the integrity of the body (9–16).

It can be assumed that working on an emergency medical team is a particular stressor. This is due to both the specifics of EMT work and various organisational factors. Multicentre studies and systematic reviews indicate that the incidence of mental disorders is significantly higher in the emergency medical personnel population than in the general population. First and foremost, paramedics are more likely to suffer from post-traumatic stress disorder (PTSD) (approximately 11% of the global population). In addition, paramedics are more likely to present with symptoms of depression and/or anxiety disorders (approximately 15%) (17, 18).

The immediate traumatic experiences resulting from daily professional practice are the most obvious source of stress. In the course of their work, paramedics regularly encounter patients whose lives and health are at risk. These are often seriously injured or dying patients. EMTs also provide medical assistance to children, which most staff perceive as a much more psychologically stressful situation. From 2023, paramedics will also be able to pronounce a patient dead if emergency medical procedures prove ineffective, which is undoubtedly a stressful event, especially when this information has to be communicated to the family present at the scene. At the same time, paramedics are, regrettably, increasingly exposed to aggression aimed at them directly. They find themselves in a difficult situation in which, on the one hand, they want to help someone in need, but on the other hand, they risk being attacked by bystanders. It all leads to cumulative exposure (repeated minor traumas combined with episodic serious events), which increases the risk of developing post-traumatic stress disorder and chronic stress. Qualitative and quantitative studies show that it is not only a single “major” event, but also cumulative exposure and lack of time to process experiences (another dispatch to an emergency) that are strong predictors of PTSD and burnout symptoms (19, 20, 21, 23).

Another stressful aspect of a paramedic’s work is handling emergency calls that are not intended for the emergency medical team within the healthcare system, such as calls from older population resulting from a lack of social care and their feelings of loneliness and abandonment (21).

Another stress factor is the organisation of work itself and the excessive workload in the 12-h system. This disrupts the circadian rhythm, making it difficult to interact with family members. This is compounded by staff shortages, overtime, a lack of transparent procedures, poor internal communication, insufficient support from superiors, and job insecurity. Reports and studies from the last decade repeatedly identify “organisational” determinants as those that most predispose to chronic stress and burnout, often even more strongly than single traumatic events (19, 20, 24, 25).

It is clear that not all paramedics react in the same way; factors such as length of service, pre-existing mental health issues, lack of social support outside of work, low professional self-efficacy, and inadequate coping mechanisms increase susceptibility to the adverse effects of stress. Paramedics are less likely to seek psychological assistance. Emotional isolation and professional standards that promote toughness can hinder adaptive coping and contribute to stress (18, 22).

In view of the above, it appears worthwhile to undertake research to assess the level of stress among active paramedics/nurses and to analyse personal, socio-demographic, and professional factors that determine stress and ways of coping in difficult situations. Knowledge of coping strategies can help take targeted preventive and corrective measures. Identifying the problem also guides leaders in the profession in shaping favourable working conditions and academic teachers in implementing curricula that prepare young people for complex professional roles.

The research seeks answers to the following research questions:

1. What stress coping strategies (Mini-COPE) predominate among EMT personnel, and how are they related to the level of perceived stress (PSS-10)?
2. Is there a relationship between self-efficacy (GSES), psychological resilience (SPP-25) and level of work engagement (UWES) and the level of perceived stress in the group of EMT personnel?
3. How do socio-demographic factors (age, gender, length of service, position, number of hours worked per month) differentiate the psychosocial functioning of emergency medical personnel?
4. What significant predictors of stress are identified in the studied group of EMT personnel?

## Research material and methods

The study covered 150 paramedics and nurses employed under various contracts (employment contract, civil law contract) at the Provincial Ambulance Service in Białystok. This institution, headquartered in Białystok, also operates ambulance stations in nearby towns, including Bielsk Podlaski, Siemiatycze, Hajnówka, Sokółka, and Mońki. Therefore, the study was addressed to paramedics and nurses in the north-eastern macro-region of Poland. The cross-sectional study was conducted between May 2025 and September 2025. The sample size or power analysis wasn’t performed—sample size was planned so that more than half of the approximately 250 employees participated in the study. The inclusion criterion for the study was having a current contract with the Provincial Ambulance Service in Białystok as a paramedic or nurse. The link to participate in the study was provided only to employees with a current contract. No exclusion criteria were defined, so anyone with a current contract could participate. The main author of the study was responsible for data collection.

A diagnostic survey was used as the research method. For this purpose, a proprietary survey questionnaire was used, containing questions about socio-demographic data and work-related issues. A total of 13 open-ended and closed-ended questions were formulated. Standardised research tools were also used, including

- *The Resilience Measurement Scale (SPP-25)* developed by Ogińska-Bulik and Juczyński. It measures persistence and determination, openness to new experiences and a sense of humour, personal coping skills and tolerance of negative emotions, tolerance of failure and treating life as a challenge, an optimistic attitude towards life and the ability to summon resources in difficult situations. The scale’s reliability, as measured by Cronbach’s alpha, is 0.89 (26). The higher the total score for each dimension (0–20), the greater the resilience in that dimension. The overall SPP-25 score can be given as a sten (i.e., standard 10) score, where scores of 1–4 indicate low resilience, 5–6 indicate moderate resilience, and 7–10 indicate high resilience (26).
- *The Utrecht Work Engagement Scale (UWES)* as adapted into Polish by Szabowska-Walaszyk, Zawadzka, and Wojtaś. The questionnaire contains 17 items with a 7-point response scale ranging from 0 (“never”) to 6 (“always/every day”). The UWES produces scores on three subscales: vigour, absorption, and dedication (27).
- *The General Self-Efficacy Scale (GSES)* by Schwarz, Jerusalem, and Juczyński is a research tool consisting of 10 questions designed to examine an individual’s general belief in their ability to cope with difficulties and obstacles. The respondents provide answers on a four-point scale. The overall score ranges from 10 to 40. A high sense of self-efficacy is perceived by those who scored 30 or more, which corresponds to the 7–10 sten score; moderate is in the range of 25–29, which corresponds to the 5–6 sten score; and low is the score of 24 and below (1–4 sten score). Its Cronbach’s alpha coefficient is 0.85. The scale is intended for testing healthy and diseased adults (28).
- *The Perceived Stress Scale (PSS-10)* measures perceived stress. It was developed by Cohen, Kamarck, and Marmelstein. It was adapted into Polish by Juczyński and Ogińska-Bulik. The scale contains 10 questions concerning stressful events, personal problems, and feelings associated with various life situations. The assessment of stress intensity, on a scale of 0–5, is based on responses to questions about irritability, nervousness, and coping with stress. The total score ranges from 0 to 40. The higher the score, the more severe the stress symptoms. The overall stress score is interpreted using a sten score, where 1–4 = low score; 5–6 = moderate score; 7–10 = high score (29).
- *The Inventory for Measuring Coping With Stress (Mini-COPE)* assesses coping skills, i.e., typical ways of reacting and feeling in situations of severe stress. Mini-COPE consists of 28 items that make up 14 scales corresponding to coping strategies: active coping, planning, positive reframing, acceptance, humour, turning to religion, seeking emotional support, seeking instrumental support, self-distraction, denial, venting, substance use, behavioural disengagement, and self-blame. The range of scores on each scale is between 0 and 3. The higher the score, the greater the intensity of the chosen strategy (30).

Respondents’ responses were recorded via Google. The survey questionnaires were combined into a single questionnaire and made available to participants via an online link. The beginning of each questionnaire was marked on the completed questionnaire. To submit the responses, all questions included in the survey had to be answered.

The raw data, saved in Excel, were downloaded for statistical analysis. Participation in the study was voluntary. The study was anonymous, and each participant could withdraw from it at any time. Participation was considered equivalent to consent to participate in the study.

### Ethics consideration

The research was carried out in accordance with the Declaration of Helsinki and Good Clinical Practice. The Bioethics Committee of the Medical University in Białystok, Poland, granted ethical approval for the study (APK.002.234.2025). Participation was voluntary, and participants were informed about the project and gave written consent.

### Statistical analysis

The analyses were performed using IBM SPSS Statistics 29. For quantitative variables, basic descriptive statistics were calculated, including arithmetic mean, median, standard deviation, minimum and maximum values, skewness, and kurtosis. Percentage distributions were presented for qualitative variables. The normality of the distributions of quantitative variables was assessed using the Shapiro–Wilk test.

The following statistical analysis methods were used to verify the research problems: Pearson’s *r* test—to assess the strength and direction of the relationship between quantitative variables; Spearman’s rho for ordinal variables; Student’s *t*-test for independent samples—to compare means in two groups, Mann–Whitney *U* test—in cases where the compared groups differed in size or did not meet the assumptions of parametric tests; the Kruskal–Wallis test—to compare more than two independent groups in the absence of parametric test assumptions; linear regression analysis using the enter method—to determine which socio-demographic and psychological variables significantly predict the level of perceived stress.

A significance level of *α* = 0.05 was adopted for all analyses. The presentation of the results also included the values of the effect sizes (*r*, *η*^2^, Cohen’s *d*, *β*), which allows for an assessment of the practical significance of the results.

## Results

### Socio-demographic characteristics of the study group

The analysis covered the socio-demographic data of 150 employees of the Provincial Ambulance Service in Białystok. The study group included 79 paramedics and 71 nurses. The mean age of the respondents was 43.17 years (SD ± 11.53), ranging from 24 to 66. The study group included 51.3% women (*n* = 77) and 48.0% men (*n* = 72). Considering their place of residence, over half of the respondents lived in large cities with a population of over 250,000 (52.7%). In terms of marital status, most were married or in a civil partnership (51.3%). Taking into account the respondents’ education, the largest group consisted of those with a master’s degree in nursing (42.7%) and a bachelor’s degree in emergency medicine (32.0%).

By job position, a significant percentage were paramedics/nurses serving as emergency medical team leaders (44.0%). Respondents who had been working in the profession for 6 to 15 years accounted for 46.7%. In terms of employment status, contract staff members predominated (65.3%). Most respondents had a single employer (67.3%). In addition, 70.0% of respondents reported working overtime. The mean number of hours worked per month was 178.67 (SD ± 32.52), ranging from 100 to 250 h. Details are presented in Table 1.

**Table 1:** Socio-demographic characteristics of the study group of emergency medical service workers (*n* = 150).

Variable	*n*	%	*M*	SD	Min.	Max.
Age			43.17	11.53	24.00	66.00
Gender						
Female	77	51.3				
Male	72	48.0				
Place of residence						
Rural area	24	16.0				
Town/city with a population of up to 50,000	42	28.0				
Town/city with a population of 51,000–100,000	5	3.3				
City with a population of 250,000 or more	79	52.7				
Marital status						
Single	38	25.3				
Divorced	35	23.3				
Married/in a relationship	77	51.3				
Education						
Bachelor’s degree in nursing	3	2.0				
Bachelor’s degree in emergency medicine	48	32.0				
Master’s degree in nursing	64	42.7				
Other master’s degree programmes, e.g., public health	4	2.7				
School of Emergency Medical Services	31	20.7				
Position						
Paramedic/nurse	39	26.0				
Paramedic/nurse—emergency medical team leader	66	44.0				
Paramedic/nurse—driver	45	30.0				
Length of service [years]						
Up to 5 years	30	20.0				
6–15 years	70	46.7				
16–25 years	37	24.7				
Over 25 years	13	8.7				
Form of employment						
Contract staff	98	65.3				
Permanent employment contract	49	32.7				
Fixed-term employment contract	3	2.0				
Shifts						
12 h	74	49.3				
24 h	75	50.0				
25 h	1	0.7				
Number of jobs						
One	101	67.3				
Two	45	30.0				
Three	4	2.7				
Overtime work						
No	45	30.0				
Yes	105	70.0				
Number of hours worked per month			178.67	32.52	100.00	250.00

*n*, number of observations; *M*, mean; SD, standard deviation; Min., minimum value; Max., maximum value.

### Assessment of the psychological resilience, work engagement, sense of self-efficacy, perceived stress levels, and coping strategies in difficult situations of respondents

Subsequently, basic descriptive statistics and the results of the Shapiro–Wilk normality test for quantitative variables used in the study were analysed. This analysis aimed to assess the normality of the distribution of the studied variables and to determine their intensity (Table 2).

**Table 2:** Basic descriptive statistics for SPP-25, UWES, GSES, PSS-10, and Mini-COPE (*n* = 150).

Dependent variable	*M*	Me	SD	Sk.	Kurt.	Min.	Max.	*W*	*p*
Overall resilience (SPP-25)	64.95	66.00	22.95	−0.49	−0.90	16.00	99.00	0.94	**<0.001**
Persistence and determination	13.08	14.00	4.68	−0.34	−1.20	5.00	20.00	0.92	**<0.001**
Openness to new experiences and sense of humour	12.85	13.00	4.59	−0.51	−0.78	3.00	20.00	0.94	**<0.001**
Personal coping skills and tolerance of negative emotions	13.10	14.00	4.76	−0.66	−0.51	2.00	20.00	0.92	**<0.001**
Tolerance of failure and treating life as a challenge	12.92	13.00	4.81	−0.43	−0.78	2.00	20.00	0.95	**<0.001**
Optimistic attitude towards life and the ability to summon resources in difficult situations	13.01	14.00	4.66	−0.45	−0.79	2.00	20.00	0.94	**<0.001**
Overall work engagement (UWES)	3.52	3.94	1.43	−0.32	−1.35	1.06	5.71	0.91	**<0.001**
Vigour	3.53	3.83	1.44	−0.25	−1.31	1.00	6.00	0.92	**<0.001**
Dedication	3.50	4.00	1.43	−0.29	−1.32	1.00	5.60	0.91	**<0.001**
Absorption	3.52	4.00	1.43	−0.38	−1.32	0.83	5.50	0.90	**<0.001**
Self-efficacy (GSES)	27.60	27.00	5.43	−0.44	−0.14	14.00	38.00	0.96	**<0.001**
Level of perceived stress (PSS-10)	22.31	22.00	7.28	−0.10	−0.97	8.00	34.00	0.96	**<0.001**
Coping strategies (Mini-COPE)									
Active coping	1.60	1.50	0.53	0.46	−0.07	0.50	3.00	0.91	**<0.001**
Planning	1.80	1.50	0.59	−0.13	−1.21	0.50	2.50	0.86	**<0.001**
Positive reframing	1.63	1.50	0.60	−0.33	−0.34	0.00	2.50	0.92	**<0.001**
Acceptance	1.62	1.50	0.61	0.06	−0.28	0.50	3.00	0.93	**<0.001**
Sense of humour	1.78	2.00	0.57	0.09	−0.53	0.50	3.00	0.93	**<0.001**
Turning to religion	1.57	1.50	0.63	0.21	−0.76	0.50	3.00	0.92	**<0.001**
Seeking emotional support	1.87	2.00	0.66	−0.43	0.38	0.00	3.00	0.93	**<0.001**
Seeking instrumental support	1.74	1.50	0.60	−0.37	0.20	0.00	3.00	0.92	**<0.001**
Self-distraction	1.63	1.50	0.48	0.23	−0.91	1.00	2.50	0.87	**<0.001**
Denial	1.52	1.50	0.51	0.14	−0.54	0.50	2.50	0.91	**<0.001**
Venting	1.54	1.50	0.55	0.70	0.23	0.50	3.00	0.88	**<0.001**
Substance use	1.56	1.75	0.77	−0.31	−0.56	0.00	3.00	0.93	**<0.001**
Behavioural disengagement	1.63	1.50	0.62	0.23	−0.47	0.50	3.00	0.93	**<0.001**
Self-blame	1.62	1.50	0.63	0.19	−0.92	0.50	3.00	0.91	**<0.001**

*M*, arithmetic mean; Me, median; SD, standard deviation; Sk., skewness; Kurt., kurtosis; Min., minimum value; Max., maximum value; *W*, Shapiro–Wilk test result; *p*, *p*-value for the Shapiro–Wilk test.

#### Psychological resilience (SPP–25)

Regarding the intensity of the analysed variables, the individual components of psychological resilience were at levels close to the mean. Persistence and determination had a mean score of 13.08 (SD = 4.68), while openness to new experiences and sense of humour had a mean score of 12.85 (SD = 4.59). Similar scores were reported for personal coping skills and tolerance of negative emotions (*M* = 13.10; SD = 4.76), tolerance of failure and treating life as a challenge (*M* = 12.92; SD = 4.81), and an optimistic attitude towards life and the ability to summon resources in difficult situations (*M* = 13.01; SD = 4.66). These results suggested a moderate level of individual resources contributing to resilience.

The mean overall psychological resilience score for the respondents was 64.95 (SD ± 22.95), ranging from 16 to 99, which indicates a varied level of resilience in the study group (Table 2). Taking the generally accepted sten scores into account, the mean score for the entire study group (64.95) falls within the 60–65 range, corresponding to a sten score of 4, indicating low psychological resilience.

#### Work and well-being (UWES)

Regarding the work engagement subscales, moderate intensity was observed across all three dimensions. Mean scores for vigour were 3.53 (SD = 1.44), dedication—3.50 (SD = 1.43), and absorption—3.52 (SD = 1.43). Similar mean and standard deviation values indicated a similar degree of intensity across all engagement dimensions.

Overall work engagement was moderate, with a mean score of 3.52 (SD ± 1.43) on a scale ranging from 1.06 to 5.71 (Table 2).

#### Self-efficacy (GSES)

Self-efficacy had a mean score of 27.60 (SD ± 5.43) (Table 2). Taking the generally accepted sten scores into account, the mean score for the entire study group falls within the range of 25–29, corresponding to a sten score of 5–6, indicating moderate self-efficacy.

#### Level of perceived stress (PSS-10)

The mean score for perceived stress in the sample was 22.31 (SD ± 7.28) (Table 2).

#### Ways of coping with stress (Mini-COPE)

With regard to stress coping strategies, the most commonly used were seeking emotional support (*M* = 1.87; SD ± 0.66), seeking instrumental support (*M* = 1.74; SD ± 0.60), sense of humour (*M* = 1.78; SD ± 0.57), and planning (*M* = 1.80; SD ± 0.59). The least common strategies were substance use (*M* = 1.56; SD ± 0.77) and denial (*M* = 1.52; SD ± 0.51), suggesting that the respondents preferred more constructive forms of coping. A moderate level of use was also recorded for venting (*M* = 1.54; SD = 0.55).

In addition, standardised scores for perceived stress (sten scores) obtained using the Perceived Stress Scale (PSS-10) were analysed. The mean sten score for the entire study group was 7.26 (SD ± 2.14), indicating elevated stress levels among paramedics/emergency nurses.

Quartile analysis revealed that half of the respondents had sten scores ranging from 5 to 9, indicating that most respondents experienced moderate to high levels of stress. Sten scores above 9 reported by some respondents indicate a very high level of perceived stress among a considerable number of respondents. Details are provided in Table 3.

**Table 3:** Basic descriptive statistics for normalised scores of perceived stress levels (*n* = 150).

Dependent variable	*M*	Me	SD	Lower quartile	Upper quartile	Min.	Max.
Level of perceived stress—normalised scores	7.26	7.00	2.14	5.00	9.0	3.00	10.00

*M*, arithmetic mean; Me, median; SD, standard deviation; Min., minimum value; Max., maximum value.

### Relationships between resilience, work engagement, self-efficacy, perceived stress levels and stress coping strategies

Pearson’s r correlation analysis was performed to assess the relationship between the analysed variables. The results indicated that the overall level of psychological resilience (SPP-25) strongly positively correlated with overall work engagement (UWES) (*r* = 0.67; *p* < 0.001) and moderately positively with self-efficacy (GSES) (*r* = 0.47; *p* < 0.001). No significant relationship was found between resilience (SPP25) and perceived stress (PSS10) (*r* = 0.05; *p* > 0.05).

Self-efficacy (GSES) remained strongly positively correlated with work engagement (UWES) (*r* = 0.55; *p* < 0.001) and weakly negatively correlated with perceived stress levels (PSS10) (*r* = −0.27; *p* < 0.001).

In terms of coping strategies (Mini-COPE), weak or moderate positive correlations were found between psychological resilience, work engagement and self-efficacy and the following strategies: active coping (*r* = 0.26; *p* < 0.01 for resilience; *r* = 0.23; *p* < 0.01 for work engagement; *r* = 0.41; *p* < 0.001 for self-efficacy), positive reframing (*r* = 0.25; *p* < 0.01; *r* = 0.21; *p* < 0.01; *r* = 0.19; *p* < 0.05), seeking emotional support (*r* = 0.18; *p* < 0.05; *r* = 0.27; *p* < 0.001; *r* = 0.33; *p* < 0.001), and seeking instrumental support (*r* = 0.23; *p* < 0.01; *r* = 0.23; *p* < 0.01; *r* = 0.16; *p* < 0.05). A moderate positive correlation was also observed between resilience and self-efficacy and self-distraction (*r* = 0.23; *p* < 0.01 and *r* = 0.25; *p* < 0.01).

It is worth noting that substance use was positively, albeit weakly, correlated with self-efficacy (*r* = 0.17; *p* < 0.05), while perceived stress was weakly positively correlated with turning to religion (*r* = 0.29; *p* < 0.001) and behavioural disengagement (*r* = 0.24; *p* < 0.01). Details in Table 4.

**Table 4:** Pearson’s *r* correlation coefficients between resilience, work engagement, self-efficacy, perceived stress levels, and coping strategies (*n* = 150).

Variable	Overall resilience	Overall work engagement	Self-efficacy	Level of perceived stress
Overall resilience (SPP-25)	–			
Overall work engagement (UWES)	**0.67** ^***^	–		
Self-efficacy (GSES)	**0.47** ^***^	**0.55** ^***^	–	
Level of perceived stress (PSS-10)	0.05	0.04	**−0.27** ^***^	–
Coping strategies (Mini-COPE)				
Active coping	**0.26** ^**^	**0.23** ^**^	**0.41** ^***^	−0.15
Planning	0.14	0.04	0.06	0.11
Positive reframing	**0.25** ^**^	**0.21** ^**^	**0.19** ^*^	0.16
Acceptance	0.15	0.05	0.12	0.04
Sense of humour	0.10	0.04	0.13	0.08
Turning to religion	0.11	0.14	0.14	**0.29** ^***^
Seeking emotional support	**0.18** ^*^	**0.27** ^***^	**0.33** ^***^	0.03
Seeking instrumental support	**0.23** ^**^	**0.23** ^**^	**0.16** ^*^	0.13
Self-distraction	**0.23** ^**^	0.11	**0.25** ^**^	0.03
Denial	0.06	0.08	0.16	0.12
Venting	0.07	−0.04	0.03	0.09
Substance use	0.14	0.09	**0.17** ^*^	0.08
Behavioural disengagement	0.12	0.15	0.11	**0.24** ^**^
Self-blame	0.14	0.02	0.04	0.16

^***^p < 0.001; ^**^p < 0.01; ^*^p < 0.05.

### Correlations between psychological variables and age, length of service, and number of hours worked

In order to assess the relationship between psychological variables (psychological resilience, self-efficacy, stress, coping strategies, work engagement) and age, length of service, and number of hours worked per month, Pearson’s r correlation analysis was again performed for quantitative variables (age, number of hours worked) and Spearman’s rho correlations for length of service because this variable was ordinal (Table 5).

**Table 5:** Pearson’s *r* and Spearman’s rho correlation coefficients between psychological variables and age, length of service, and number of hours worked (*n* = 150).

Variable	Age	Number of hours worked per month	Length of service [years]
Overall resilience (SPP-25)	**−0.58** ^***^	0.06	**−0.52** ^***^
Overall work engagement (UWES)	**−0.56** ^***^	0.02	**−0.61** ^***^
Self-efficacy (GSES)	**−0.43** ^***^	**0.26** ^**^	**−0.41** ^***^
Level of perceived stress (PSS-10)	0.01	**−0.26** ^**^	0.01
Coping strategies (Mini-COPE)			
Active coping	−0.15	**0.37** ^***^	**−0.19** ^*^
Planning	−0.13	0.13	**−0.19** ^*^
Positive reframing	**−0.27** ^***^	0.07	**−0.32** ^***^
Acceptance	**−0.18** ^*^	**0.17** ^*^	−0.13
Sense of humour	**−0.17** ^*^	**0.24** ^**^	−0.16
Turning to religion	0.02	0.11	0.01
Seeking emotional support	**−0.23** ^**^	0.07	**−0.26** ^**^
Seeking instrumental support	**−0.22** ^**^	0.05	**−0.30** ^***^
Self-distraction	**−0.23** ^**^	0.11	**−0.23** ^**^
Denial	0.05	0.01	0.00
Venting	0.04	0.07	0.09
Substance use	0.03	−0.09	0.02
Behavioural disengagement	−0.04	0.06	−0.11
Self-blame	0.02	0.05	−0.08

For the variable length of service, Spearman’s rho correlation coefficient values were reported; in other cases, Pearson’s *r* coefficient values were reported.

^***^p < 0.001; ^**^p < 0.01; ^*^p < 0.05.

The results revealed significant, strong negative correlations between age and length of service with psychological resilience (*r* = −0.58; *p* < 0.001 for age; rho = −0.52; *p* < 0.001 for length of service), and overall work engagement (*r* = −0.56; *p* < 0.001; rho = −0.61; *p* < 0.001). Additionally, for both variables, significant and moderate negative correlations were found with self-efficacy (*r* = −0.43; *p* < 0.001 for age; rho = −0.41; *p* < 0.001 for length of service). This suggests that older paramedics and those with longer professional experience had lower levels of resilience, work engagement, and self-efficacy.

Furthermore, age and length of service correlated weakly or moderately negatively with several stress coping strategies. In both cases, significantly lower scores were found for positive reframing (*r* = −0.27; *p* < 0.001 for age; rho = −0.32; *p* < 0.001 for length of service), seeking emotional support (*r* = −0.23; *p* < 0.01; rho = −0.26; *p* < 0.01), seeking instrumental support (*r* = −0.22; *p* < 0.01; rho = −0.30; *p* < 0.001), and self-distraction (*r* = −0.23; *p* < 0.01; rho = −0.23; *p* < 0.01). Additionally, age correlated weakly negatively with acceptance (*r* = −0.18; *p* < 0.05) and sense of humour (*r* = −0.17; *p* < 0.05), while length of service correlated negatively with active coping (rho = −0.19; *p* < 0.05) and planning (rho = −0.19; *p* < 0.05).

With regard to the number of hours worked per month, a moderate positive correlation was found with active coping (*r* = 0.37; *p* < 0.001), acceptance (*r* = 0.17; *p* < 0.05), and sense of humour (*r* = 0.24; *p* < 0.01), and weak positive correlations with self-efficacy (*r* = 0.26; *p* < 0.01). Importantly, the number of work hours weakly negatively correlated with the level of perceived stress (*r* = −0.26; *p* < 0.01), indicating that people working more hours per month experienced lower stress levels (Table 5).

### Comparative analysis of psychological variables by gender of the respondents

Student’s *t*-test for independent samples was conducted to assess differences between women and men in psychological variables. When Levene’s test was significant, Welch’s correction was applied to the reported values (Table 6).

**Table 6:** Comparison of psychological variables between women and men (*n* = 149).

Dependent variable	Women (*n* = 77)		Men (*n* = 72)		*t*	*df*	*p*	Cohen’s *d*
	*M*	*SD*	*M*	*SD*				
Overall resilience (SPP-25)	70.58	20.60	59.33	23.94	3.07^a^	140.47	**0.003**	0.51
Overall work engagement (UWES)	3.86	1.09	3.15	1.65	3.10^a^	121.86	**0.002**	0.51
Self-efficacy (GSES)	27.04	4.77	28.22	6.07	−1.33	147	0.186	0.22
Level of perceived stress (PSS-10)	23.86	7.06	20.58	7.19	2.80	147	**0.006**	0.46
Coping strategies (Mini-COPE)								
Active coping	1.61	0.43	1.58	0.63	0.38^a^	124.11	0.701	0.06
Planning	1.84	0.67	1.76	0.50	0.84^a^	140.16	0.401	0.14
Positive reframing	1.69	0.59	1.57	0.62	1.20	147	0.232	0.20
Acceptance	1.59	0.67	1.64	0.54	−0.48^a^	143.69	0.630	0.08
Sense of humour	1.77	0.58	1.78	0.56	−0.05	147	0.957	<0.01
Turning to religion	1.62	0.65	1.51	0.59	1.14	147	0.257	0.19
Seeking emotional support	1.94	0.60	1.79	0.72	1.33	147	0.185	0.22
Seeking instrumental support	1.89	0.52	1.57	0.62	3.40	147	**<0.001**	0.56
Self-distraction	1.69	0.49	1.56	0.46	1.69	147	0.093	0.28
Denial	1.61	0.53	1.42	0.47	2.27	147	**0.025**	0.37
Venting	1.58	0.61	1.49	0.47	1.02	147	0.308	0.17
Substance use	1.77	0.67	1.33	0.81	3.69	147	**<0.001**	0.61
Behavioural disengagement	1.72	0.64	1.54	0.60	1.77	147	0.079	0.29
Self-blame	1.75	0.72	1.47	0.50	2.73^a^	135.42	**0.007**	0.44

*n*, number of observations; *M*, arithmetic mean; SD, standard deviation; *t*, *t*-statistic value; df, degrees of freedom; *p*, statistical significance.

a Levene’s test proved to be statistically significant, and the result was reported with Welch’s correction.

The analysis showed that women scored significantly higher on the overall level of psychological resilience (SPP-25) (*M* = 70.58; SD ± 20.60) compared to men (*M* = 59.33; SD ± 23.94), *p* = 0.003. The effect size was moderate. A similar finding was reported for overall work engagement: women demonstrated higher levels of engagement (*M* = 3.86; SD ± 1.09) than men (*M* = 3.15; SD ± 1.65), *p* = 0.002, with a moderate effect size as well.

No significant differences were found between women (*M* = 27.04; SD = 4.77) and men (*M* = 28.22; SD = 6.07), *p* = 0.186, in self-efficacy (GSES). However, the level of perceived stress (PSS-10) was significantly higher among women (*M* = 23.86; SD ± 7.06) than among men (*M* = 20.58; SD ± 7.19), *p* = 0.006, but the effect size was small.

In terms of coping strategies (Mini-COPE), significant differences between women and men were observed in four dimensions. Women sought instrumental support more often than men (*M* = 1.89; SD ± 0.52 vs. *M* = 1.57; SD ± 0.62), *p* < 0.001, and more often reported substance use (*M* = 1.77; SD ± 0.67 vs. *M* = 1.33; SD ± 0.81), *p* < 0.001. In both cases, significant effect sizes were observed. Additionally, women scored higher on denial (*M* = 1.61; SD ± 0.53) compared to men (*M* = 1.42; SD ± 0.47), *p* = 0.025, and self-blame (*M* = 1.75; SD ± 0.72 vs. *M* = 1.47; SD = 0.50), *p* = 0.007. However, the effect sizes of these differences were small.

For the remaining coping strategies, the differences between women and men did not reach statistical significance (Table 6).

### Comparison of psychological variables in groups by job position

The Kruskal–Wallis test was used to compare groups by job position. The three analysed groups included paramedics/nurses (*n* = 39), emergency medical team leaders (*n* = 66), and paramedics/nurses—drivers (*n* = 45). The results of the analysis are presented in Table 7.

**Table 7:** Comparison of psychological variable scores in groups by job position—Kruskal–Wallis test (*n* = 150).

Dependent variable	Paramedic/nurse (*n* = 39)		Team leader (*n* = 66)		Driver (*n* = 45)		*H*(2)	*p*	*η* ^2^
	*M*	SD	*M*	SD	*M*	SD			
Overall resilience	67.18	23.78	63.45	21.87	65.22	24.11	1.03	0.598	<0.01
Overall work engagement	3.72	1.08	3.46	1.34	3.43	1.78	0.28	0.871	<0.01
Self-efficacy	26.23	4.52	27.64	4.55	28.73	6.99	9.0	**0.011**	0.05
Level of perceived stress	27.44	4.60	21.29	7.48	19.38	6.71	27.93	**<0.001**	0.18
Stress coping strategies									
Active coping	1.59	0.40	1.65	0.51	1.52	0.65	2.49	0.288	<0.01
Planning	1.96	0.64	1.71	0.58	1.79	0.54	4.00	0.135	0.01
Positive reframing	1.76	0.48	1.63	0.64	1.52	0.63	2.69	0.260	<0.01
Acceptance	1.62	0.60	1.69	0.71	1.51	0.42	2.11	0.348	<0.01
Sense of humour	1.72	0.56	1.83	0.55	1.76	0.61	1.52	0.467	<0.01
Turning to religion	1.79	0.59	1.55	0.69	1.42	0.50	7.30	**0.026**	0.04
Seeking emotional support	2.09	0.41	1.80	0.74	1.77	0.67	6.84	**0.033**	0.03
Seeking instrumental support	2.01	0.57	1.7	0.64	1.57	0.46	13.28	**0.001**	0.08
Self-distraction	1.62	0.40	1.67	0.53	1.59	0.47	1.13	0.569	<0.01
Denial	1.49	0.58	1.7	0.46	1.29	0.42	17.69	**<0.001**	0.11
Venting	1.64	0.71	1.55	0.46	1.42	0.50	3.21	0.201	<0.01
Substance use	1.71	0.58	1.80	0.80	1.09	0.64	26.12	**<0.001**	0.16
Behavioural disengagement	1.82	0.48	1.7	0.72	1.37	0.47	16.35	**<0.001**	0.10
Self-blame	1.92	0.77	1.57	0.57	1.42	0.49	11.98	**0.003**	0.07

*n*, number of observations; *M*, arithmetic mean; Me, median; SD, standard deviation; *H*(2), *t*-statistic value with degrees of freedom; *p*, statistical significance, *η*^2^, effect size indicator.

The analysis showed significant differences in self-efficacy (GSES) (*p* = 0.011) and perceived stress (PSS-10) (*p* < 0.001). The effect size was moderate for self-efficacy and strong for stress levels. Additional *post hoc* comparisons with the Bonferroni correction (Table 8) showed that self-efficacy was significantly higher among drivers (*M* = 28.73; SD ± 6.99) than among paramedics/nurses (*M* = 26.23; SD ± 4.52), *p* = 0.009. In terms of stress levels, paramedics/nurses scored higher (*M* = 27.44; SD ± 4.60) than both team leaders (*M* = 21.29; SD ± 7.48), *p* < 0.001, and drivers (*M* = 19.38; SD ± 6.71), *p* < 0.001.

**Table 8:** Results of *post hoc* comparisons with the Bonferroni correction for significant size effects (*n* = 150).

Dependent variable	Compared pairs	*p* ^a^
Self-efficacy	Paramedic/nurse vs. Team leader	0.472
	Paramedic/nurse vs. Driver	**0.009**
	Team leader vs. Driver	0.177
Level of perceived stress	Driver vs. Team leader	0.863
	Driver vs. Paramedic/nurse	**<0.001**
	Team leader vs. Paramedic/nurse	**<0.001**
Turning to religion	Driver vs. Team leader	0.604
	Driver vs. Paramedic/nurse	**0.021**
	Team leader vs. Paramedic/nurse	0.268
Seeking emotional support	Driver vs. Team leader	1.00
	Driver vs. Paramedic/nurse	**0.038**
	Team leader vs. Paramedic/nurse	0.111
Seeking instrumental support	Driver vs. Team leader	0.210
	Driver vs. Paramedic/nurse	**0.001**
	Team leader vs. Paramedic/nurse	0.081
Denial	Driver vs. Paramedic/nurse	0.172
	Driver vs. Team leader	**<0.001**
	Paramedic/nurse vs. Team leader	0.153
Substance use	Driver vs. Paramedic/nurse	**0.001**
	Driver vs. Team leader	**<0.001**
	Paramedic/nurse vs. Team leader	1.00
Behavioural disengagement	Driver vs. Team leader	**0.006**
	Driver vs. Paramedic/nurse	**<0.001**
	Team leader vs. Paramedic/nurse	0.650
Self-blame	Driver vs. Team leader	0.807
	Driver vs. Paramedic/nurse	**0.002**
	Team leader vs. Paramedic/nurse	**0.028**

a Bonferroni correction for multiple comparisons was applied.

With regard to coping strategies (Mini-COPE), significant differences were found for substance use (*p* < 0.001; strong effect), seeking instrumental support (*p* = 0.001; moderate effect), denial (*p* < 0.001; moderate effect), and behavioural disengagement (*p* < 0.001; moderate effect), self-blame (*p* = 0.003; moderate effect), turning to religion (*p* = 0.026; weak effect), and seeking emotional support (*p* = 0.033; weak effect).

Additional post hoc comparisons identified which groups differed significantly in terms of coping strategies (Table 8):

- Regarding turning to religion, paramedics/nurses scored significantly higher than drivers (*p* = 0.021).
- Seeking emotional and instrumental support was more common among paramedics/nurses than among drivers (*p* = 0.038 and *p* = 0.001).
- In terms of denial, team leaders scored higher than drivers (*p* < 0.001).
- Substance use was significantly more frequent among both paramedics/nurses (*p* = 0.001) and team leaders (*p* < 0.001) compared to drivers.
- Behavioural disengagement was more frequent among paramedics/nurses than among drivers (*p* < 0.001), as well as among team leaders compared to drivers (*p* = 0.006).
- In the case of self-blame, paramedics/nurses scored higher than drivers (*p* = 0.002) and team leaders (*p* = 0.028).

For the remaining variables, the differences between the groups were not statistically significant.

### Multiple regression model predicting the level of perceived stress

To identify factors significantly associated with perceived stress, a linear regression model was constructed using the enter method. The model included psychological and socio-demographic variables shown in previous analyses to be significantly associated with stress levels: gender, paramedic/nurse status, hours worked per month, self-efficacy, turning to religion, and behavioural disengagement. The results of the analysis are presented in Table 9.

**Table 9:** Results of linear regression predicting the level of perceived stress (*n* = 150).

	Unstandardised regression coefficient (*B*)	Standard error (SE)	Standardised regression coefficient (Beta)	Student’s *t* test result (*t*)	Analysis of variance result (*F*)
*F*(6;142) = 11.61; *p* < 0.001; *R*^2^_adj._ = 0.301					
(Constant)	30.90	3.62		8.53	**<0.001**
Gender	0.35	1.10	0.02	0.32	0.750
Paramedic/nurse	4.81	1.30	0.29	3.72	**<0.001**
Number of hours worked per month	−0.05	0.02	−0.21	−2.92	**0.004**
Self-efficacy	−0.29	0.10	−0.21	−2.94	**0.004**
Turning to religion	2.70	0.95	0.23	2.85	**0.005**
Behavioural disengagement	1.24	0.94	0.11	1.31	0.193

Dependent variable: Level of perceived stress. *B*, unstandardised regression coefficient; SE, standard error; Beta, standardised regression coefficient; *t*, Student’s *t* test result; *F*, analysis of variance result; *R*^2^adj., adjusted *R*-squared.

The constructed model was statistically significant and well-fitted to the data: *F*(6;142) = 11.61; *p* < 0.001. The variables in the model explained 30.1% of the variance in perceived stress (*R*^2^_adj._ = 0.301).

Regression coefficient analysis indicated that the following were significant predictors of stress levels:

- Paramedic/nurse—persons performing this job had significantly higher stress levels compared to other groups (*B* = 4.81; *β* = 0.29; *p* < 0.001).
- Number of hours worked per month—a higher number of working hours was significantly associated with lower stress levels (*B* = −0.05; *β* = −0.21; *p* = 0.004).
- Self-efficacy—a higher level of self-efficacy was associated with lower stress levels (*B* = −0.29; *β* = −0.21; *p* = 0.004).
- Turning to religion—more frequent use of this coping strategy was significantly associated with higher stress levels (*B* = 2.70; *β* = 0.23; *p* = 0.005).

Other variables, such as gender (*B* = 0.35; *β* = 0.02; *p* = 0.750) and behavioural disengagement (*B* = 1.24; *β* = 0.11; *p* = 0.193), did not reach statistical significance, suggesting that their impact on stress levels in the analysed model is limited.

## Discussion

Preventing the adverse effects of stress and coping with them are prerequisites for maintaining good health and high work efficiency. Finding effective ways to cope with difficulties depends largely on one’s sense of self-efficacy and overall psychological resilience. When faced with a stressful situation, coping strategies are deployed, which, according to Lazarus and Folkman (30), can serve a task-oriented (instrumental, problem-oriented) function and a self-regulatory function, serving to reduce unpleasant tension and alleviate negative emotional states.

Many of the articles referenced nowadays address strategies for coping with stress among paramedics and nurses working in emergency medical teams (30, 33–36). This research, based on the Mini-COPE, has shown that coping with stress primarily involves seeking emotional and instrumental support. Another important way of responding to stress is planning. When interpreting selected strategies in line with the Mini-Cope authors’ conceptualisations, it should be emphasised that active coping and planning are problem-focused strategies, while seeking emotional support is emotion-focused (37). At the same time, planning and seeking instrumental support are adaptive strategies aimed at reducing perceived stress.

The choice of such strategies may indicate a willingness to actively seek solutions rather than avoid the problem, as well as a constructive approach to stressful situations among the respondents. In 2018, Kowalczuk and Krajewska-Kułak (30) conducted a comparable study among medical personnel on coping strategies. The authors also demonstrated that the most frequently used strategies were those focused on emotions and the problem. A 2020 study by Skórzewska (31) also indicated that stress reduction occurs through seeking emotional and instrumental support, while avoidance strategies and self-blame were less common. A study among paramedics in Spain conducted by Garcia’s team in 2022 (32) indicates that humour, social support, and planning are the most effective methods of coping with stress, which also help counter its adverse effects, such as burnout.

A similar area of research was addressed by Almutairi et al. in 2020 (23). The authors surveyed 270 emergency medical service professionals using a different tool (i.e., Coping Methods Checklist, CMC) than the one used in this study. Their results indicated that the most common strategies for coping with stress included talking to colleagues (87.4%), looking forward to being off duty (82.6%), and thinking about the positives of their work (81.1%). Notably, in the aforementioned study and in the present study, more constructive coping styles, such as problem-focused or adaptive coping, predominate, and fewer respondents report ineffective coping strategies, such as venting, denial, or behavioural disengagement, which are associated with psychological discomfort.

The authors of this study also demonstrated that humour was the third most common coping strategy. According to Juczyński, a sense of humour and venting are less effective strategies for coping with stress, although they can be instrumental in some situations (37).

In analysing perceived stress levels, this study found that the employees of the Provincial Ambulance Service in Białystok experience moderate to high occupational stress. In addition, the results showed that the respondents had low psychological resilience, moderate work engagement, and moderate self-efficacy. Furthermore, stress levels were higher among paramedics and nurses than among team leaders and drivers, but lower among those who worked longer hours per month. These findings are not surprising, as similar results were reported by Bardhan and Byrd (2023) (38). Their study showed that emergency service personnel, including paramedics, are among the groups particularly vulnerable to chronic stress related to the job. In addition, they are at risk of exposure to traumatic situations and unpredictable events. Spychała et al. conducted similar research in 2023 (39), assessing work-related stress among paramedics during the challenging circumstances of the COVID-19 pandemic. Undeniably, the epidemiological situation and the increased number of interventions significantly increased perceived stress, which translated into perceived burnout. Piotrowski obtained similar results in 2021 (40), examining paramedics both before and during the COVID-19 pandemic. At that time, employees reported not only an increase in subjectively perceived stress but also stress stemming from the burden on the healthcare system.

Also noteworthy is the meta-analysis by Petrie et al. (2018). The researchers analysed 27 studies involving a total of 30,878 emergency medical service professionals. It was noted that stress levels among them were so high that 11% of respondents developed post-traumatic stress disorder, 15% showed symptoms of depression, and 27% experienced general stress (17).

Literature reports and the present study findings prove that the profession of paramedic/emergency nurse is associated with a high risk of stress.

The researchers behind this study also demonstrated that the roles of team leader or ambulance driver are associated with lower stress levels than those of paramedic and nurse. It appears that these differences are primarily due to professional experience. In addition, ambulance drivers have slightly different tasks. They may not always provide direct medical assistance and are less exposed to direct contact with emergency and traumatic situations. In 2018, Austin et al. (41) studied secondary traumatic stress among paramedics and found that it is the direct involvement of personnel in medical incidents that causes the highest levels of emotional stress, especially when compared to those performing technical or administrative roles. The results of this study are further corroborated by the aforementioned work by Piotrowski (40). The researcher emphasises that stress among paramedics and nurses also stems from their responsibility for human life and the risk of committing medical errors.

An interesting finding from the analysis presented here is that individuals who work longer hours each month experience less stress. This conclusion is not supported by the literature. Both Austin and Zhu suggest that long working hours increase stress and accelerate burnout (41, 42). Perhaps those who choose to work longer hours per month have better coping strategies and a higher sense of self-efficacy. Austin et al. suggest that paramedics with greater psychological resilience are less susceptible to the negative effects of shift work and occupational stressors (41). In turn, Bardhan and Byrd (38) highlight the impact of organisational factors. Professionals with greater work engagement and better support from their superiors are more likely to take on additional shifts, though this is not directly related to the professionals’ own resources.

The present study has shown that the level of psychological resilience in the study group is low. This is consistent with Piotrowski’s findings, which showed that the overall psychological resilience among paramedics is lower than that of the general population, despite some paramedics having a moderate level of resilience (40). On the other hand, respondents with higher psychological resilience were characterised by greater work engagement and higher self-efficacy. They chose active coping strategies, such as seeking emotional and instrumental support. The aforementioned Piotrowski demonstrated that higher levels of resilience among paramedics were associated with a greater sense of meaning in their work and contributed to better emotional functioning and lower stress levels (40). Similar results were reported by Losoi et al. (43), who studied these parameters among healthcare professionals. Their research clearly indicated that more resilient individuals tend to display positive emotions at work, are more committed to their tasks, and cope better with stress. Garcia et al., on the other hand, found that people with lower resilience were more likely to choose avoidance or emotional strategies, such as denial or self-blame (32).

The results of this study indicate moderate work engagement and self-efficacy in the study group. A 2023 study by Zhu et al. (42) among nurses found that work engagement is moderate and strongly depends on perceived organisational support and interpersonal relationships in the workplace. Additionally, engagement decreases with increasing stress and burnout, which confirms the relationship observed in this study. Piotrowski notes that higher efficacy is associated with lower stress levels and better professional functioning (40). A moderate level of efficacy in the study group may therefore be an intermediary factor between stress and psychological resilience.

The present study found that lower levels of psychological resilience, work engagement, and self-efficacy characterised older paramedics and nurses with longer work experience. It may be the result of long-term occupational stress or related to burnout. Similar correlations have been reported in the literature. Spoorthy et al. (2021) (44) found, in a study involving paramedics and nurses, that psychological resilience and work engagement decrease with age and length of service. The authors explain this by chronic exposure to stress and limited opportunities for psychological recovery. In turn, Chow’s team (45), analysing a group of nurses, noted that self-efficacy decreases with age. This is particularly pronounced among individuals experiencing emotional fatigue, further exacerbated by poor management and a lack of organisational support. Similar conclusions were presented by Leszczyński (46), who also studied paramedics and nurses. It was observed that younger participants in the study demonstrated higher psychological resilience and greater engagement. Those with longer service demonstrated emotional exhaustion and a decline in their sense of purpose in their work.

Gender also differentiated the surveyed emergency medical service personnel in terms of their psychological resources and work engagement. Women had higher resilience scores and were more engaged in their work, but at the same time, they were more stressed and more likely to use less effective strategies (denial, self-blame, or substance use). Similar findings were also reported by Leszczyński (46). The female participants demonstrated higher psychological resilience and engagement, but at the same time, they reported symptoms of stress more frequently and used emotional coping strategies, such as seeking support or avoiding the problem. Garcia (32) revealed that women more often than men use emotion-focused strategies, including seeking support and positive reframing, while men more often choose avoidance or task-focused strategies. The authors point out that these differences may result from differences in socialisation patterns and in the ways emotions are expressed (32). In turn, Zielińska-Więczkowska (47), who studied nurses and paramedics, confirmed that although women achieved higher scores in terms of empathy and engagement, they were more likely to resort to maladaptive strategies and substance abuse. This may be due to emotional overload and insufficient organisational support in the workplace.

### Research limitations

The study certainly has limitations. Firstly, it was a cross-sectional study based solely on self-assessment. Although the standardised research tools used in this study are sensitive instruments designed to detect various behaviours and attitudes, they all focus on respondents’ subjective feelings rather than objective criteria, which creates the risk of false-positive results. Secondly, the study group was mostly from a selected macro-region, which limits generalisation of the results to the entire Polish population. Despite these limitations, the results of this study can serve as a starting point for further research on stress risk factors and effective coping strategies. This also indicates the objectives and tasks that senior management must focus on to shape appropriate working conditions and develop strategies to address difficult situations. The results also indicate the need for targeted vocational training for paramedics and nurses in this area.

## Conclusion

The research provided the following conclusions:

1. The emergency medical service personnel surveyed were characterised by moderate or high perceived occupational stress, low psychological resilience, and moderate work engagement and self-efficacy.
2. Prevalent coping strategies included emotion-focused approaches, such as seeking emotional support, and problem-focused and adaptive approaches, such as seeking instrumental support, planning, and a sense of humour.
3. High psychological resilience was associated with greater work engagement and self-efficacy. These individuals preferred active coping, positive reframing, and seeking emotional and instrumental support.
4. Paramedics and senior nurses with greater work experience were characterised by lower psychological resilience, work engagement, and self-efficacy.
5. Respondents differed by gender in terms of their psychological resources and work engagement. Women performed significantly better in this respect. However, the level of perceived stress was higher in women than in men, who mainly chose coping strategies which entail psychological discomfort and are ineffective.
6. The stress predictors included working as a paramedic/nurse, number of hours worked per month, sense of self-efficacy, and turning to religion.

The results of the present study and analysis of the available literature indicate that stress is a significant and indispensable element of the work of emergency medical teams. The above study highlights the need to counteract stress, especially among paramedics and nurses. It is essential to identify staff needs, paying particular attention to older individuals and those with longer service, to provide professional assistance in crises, and to implement preventive measures.

This study may provide a foundation for the development of comprehensive training programmes on effective stress management for students at the academic level and emergency service personnel affected by chronic occupational stress.

## Data Availability

The original contributions presented in the study are included in the article/supplementary material, further inquiries can be directed to the corresponding author/s.
